# Higher Levels of Secretory IgA Are Associated with Low Disease Activity Index in Patients with Reactive Arthritis and Undifferentiated Spondyloarthritis

**DOI:** 10.3389/fimmu.2017.00476

**Published:** 2017-04-27

**Authors:** Fabián Salas-Cuestas, Wilson Bautista-Molano, Juan M. Bello-Gualtero, Ivonne Arias, Diana Marcela Castillo, Lorena Chila-Moreno, Rafael Valle-Oñate, Daniel Herrera, Consuelo Romero-Sánchez

**Affiliations:** ^1^Faculty of Medicine, Universidad Militar Nueva Granada, Bogotá, Colombia; ^2^Unit of Oral Basic Investigation-UIBO, School of Dentistry, Universidad El Bosque, Bogotá, Colombia; ^3^Department of Rheumatology and Immunology, Hospital Militar Central, Bogotá, Colombia; ^4^School of Medicine, Instituto de Genética Humana, Pontificia Universidad Javeriana, Bogotá, Colombia

**Keywords:** spondyloarthritis, ankylosing spondylitis, secretory immunoglobulin A, immunoglobulin A, HLA-B27, severity of illness index

## Abstract

**Introduction:**

Both reactive arthritis (ReA) and undifferentiated spondyloarthritis (uSpA) belong to the group of autoinflammatory diseases called spondyloarthritis (SpA). Hypotheses have been proposed about a relationship between the intestinal mucosa and inflammation of joint tissues. The role of immunoglobulin IgA or secretory immunoglobulin A (SIgA) in the inflammatory and/or clinical activity of patients with SpA remains poorly understood.

**Objective:**

To evaluate the status of total IgA and SIgA, and the association among the levels of SIgA, IgA, IgA anti-*Chlamydia trachomatis*, and anti-*Shigella* spp. with the disease activity measures, erythrocyte sedimentation rate (ESR) and C-reactive protein (CRP) levels, was compared in a cohort of patients with ReA and uSpA and healthy subjects.

**Methods:**

This was a cross-sectional study. The serum concentrations of SIgA, IgA anti-*C. trachomatis*, anti-*Shigella* spp., and total IgA were measured. Disease activity was measured in each patient by means of Bath Ankylosing Spondylitis Disease Activity Index (BASDAI) and Ankylosing Spondylitis Disease Activity Score (ASDAS). Statistical analysis did include as bivariate evaluation, comparisons by Student’s *t*-test, Kruskal–Wallis test, and *U* Mann–Whitney test, with a multivariate evaluation by principal components analysis (PCA). A correlation analysis was carried out using the Pearson correlation coefficient and a linear regression models. All analysis were made using Stata version 11.2^®^ for Windows, R V3.3.21. Statistical significance was defined a *p*-value <0.05.

**Results:**

In all, 46 patients (78.2% men; mean age, 34.8 ± 12.3 years) and 53 controls (41% men; mean age, 32 ± 11.4 years) were included in the study. The mean serum levels of SIgA were higher in SpA patients than in healthy subjects (*p* < 0.001). Only SIgA levels correlated with disease activity: BASDAI (*r* = −0.42, *p* = 0.0046), ASDAS-CRP (*r* = −0.37, *p* = 0.014), and ASDAS-ESR (*r* = −0.45, *p* = 0.0021). The negative correlation between SIgA and all activity indices was higher in HLA-B27-positive patients (BASDAI *r* = −0.70, *p* = 0.0009, ASDAS-CRP *r* = −0.58, *p* = 0.0093, and ASDAS-ESR *r* = −0.57, *p* = 0.0083). The PCA showed three factors: the first component was constituted by variables referred as clinical activity measures, the second did include the serological activity markers, and the last component was compounded by age and symptoms time.

**Conclusion:**

Elevated serum levels of SIgA were found to be related with low disease activity in patients with ReA and uSpA.

## Introduction

Spondyloarthritis (SpA) comprises a group of rheumatic diseases including ankylosing spondylitis (AS), arthritis/spondylitis with inflammatory bowel disease (IBD), psoriatic arthritis (PsA), reactive arthritis (ReA), and undifferentiated spondyloarthritis (uSpA). SpA describes phenotypes with shared genetic, physiopathological, and clinical characteristics (1–4). The clinical course of the disease varies, with important prognostic implications related to structural damage (5, 6). ReA is a spondyloarthropathic disorder characterized by inflammation of the joints and tissues occurring after gastrointestinal or genitourinary infections, where the arthritic process is generated about 3–4 weeks postinfectious, because of the short time of symptoms (days or weeks), many extra-articular features such as gastrointestinal involvement may not be clearly identified in the clinical setting. Even this manifestation could be asymptomatic. The most commonly associated pathogens are *Salmonella* spp., *Shigella* spp., *Campylobacter* spp., *Yersinia* spp., and *Chlamydia trachomatis* (7–9).

Several studies have shown the presence of bacterial antigens in joints of patients with ReA (10). Some of the mechanisms proposed to explain this fact consider a macrophages that have phagocytosed bacteria in the intestinal lumen travel to joints attracted by adhesion molecules (7) and some intestinal antigens have been detected in peripheral blood for almost 4 years after initial enteric infection and have also been shown to persist for prolonged periods in lymph nodes, liver, spleen, lungs, and joints causing inflammation in these areas (11). In addition, this bacteria invades the intestinal mucosa generating different pulses of infection allowing the antigen to travel through the bloodstream and spread in the joints (11), causing a local inflammatory response where the antigen triggers a T CD8 response, with a CD4 T response, possibly differentiating to LT Th2, which contributes to the persistence of the antigen in the joint (12).

In the mid-1980s, Mielants et al. reported that there were inflammatory signs in seronegative patients with predominantly peripheral arthritis, by examining the histology of the ileal mucosa and the ileocecal valve, and the inflammation was independent of the status of the HLA-B27 allele (13). Furthermore, they showed that there was a correlation of these inflammatory lesions in the gut mucosa with joint inflammation (14, 15). The authors suggested that the inflammatory response of the intestinal mucosa could be related to the clinical recurrences and flares of SpA.

Many hypotheses then emerged to account for the proposed relationship between mucosal and joint inflammation (16–24). The intestinal mucosa exhibits multiple mutually complementary strategies for maintaining bacteria confined to the intestinal lumen. These strategies also participate in regulating the composition and density of the lumen (25). Similarly, the intestinal microbiota regulates the development of the immune system of the intestinal mucosa, reflecting a close and constant interaction between these two players (26–29).

In the immune system of the mucosa, Peyer patches have been studied as the gut-associated lymphoid tissue (GALT) with the highest generation of plasma cells, which produce IgA dimers (dIgA) in response to activation by intestinal antigens (30). Secretory immunoglobulin A (SIgA) consists of an IgA dimer (mainly the subclass IgA2 in humans) joined to a J-chain and the secretory component (SC). SIgA mainly functions in the intestinal lumen after an active process of trans-epithelial transport called transcytosis, which is mediated and regulated by the polymeric immunoglobulin receptor (pIgR) (25, 31, 32). Monomer immunoglobulin A, mainly produced by the plasma cells of the bone marrow, is predominantly observed in the serum (33).

In 1973, Veys and van Laere reported elevated serum levels of IgG, IgM, and IgA in patients with AS compared to the healthy population. They also reported that there were no differences between patients with peripheral compromise, when the time of evolution of the disease was taken into account (except for IgG in patients with 10 years or more of evolution) (34). In 1980, Granfors et al. reported the persistence of specific IgA and IgG antibodies against *Yersinia enterocolitica* in patients with arthritis after acute infection with this microorganism (35). Subsequently, in 1986, Granfors reported that serum concentrations of total SIgA and specific SIgA against *Y. enterocolitica* were greater in patients who developed ReA (18). The correlation among serum levels of total IgA with C-reactive protein (CRP), clinical signs, and the activity of the disease in patients with AS was observed by other researchers in the 1980s (36–40). However, other authors did not find a positive correlation between IgA and CRP (41). Finally, in 1996, Wendling and collaborators evaluated the serum levels of SIgA, total IgA, and free SC in patients with AS and found higher levels compared to healthy controls, regardless of HLA-B27 status and peripheral or axial involvement (42).

Currently, it is being investigated whether the serum levels of SIgA reflect the over-activation of the mucosal immune system, and if the antigenic stimuli in the mucosa are responsible for inducing and maintaining the inflammatory response in SpA; it is proposed that SIgA could correlate with the inflammatory and disease activity in patients with ReA and uSpA. Thus, SIgA could be clinically useful in the future for patient monitoring. Based on this premise, we investigated the correlation of SIgA, total IgA, IgA anti-*C. trachomatis*, and IgA anti-*Shigella* spp. with disease activity measurements and the erythrocyte sedimentation rate (ESR) and serum levels CRP (43, 44).

## Materials and Methods

### Study Design

A Descriptive and Analytic study was carried out, which evaluated the status of total IgA and SIgA, and the association between serum levels of SIgA, total IgA, and specific IgA anti-*Shigella* spp. and IgA anti-*C. trachomatis* with disease activity measurements [Bath Ankylosing Spondylitis Disease Activity Index (BASDAI), Ankylosing Spondylitis Disease Activity Score (ASDAS)-CRP, ASDAS-ESR], serum levels of ESR and CRP, and clinical manifestations in patients with ReA and uSpA, compared with healthy subjects who were attending the outpatient rheumatology service in the Hospital Militar Central in Bogotá, Colombia.

### Study Population

Patients and controls were selected in the outpatient office by rheumatologists, and the following inclusion criteria were applied: at least 18 years old and fulfilling the European Spondyloarthropathy Study Group (ESSG) classification criteria (45). All patients had nonsteroidal anti-inflammatory drug (NSAID) for treatment, the therapeutic approach of SpA is similar with regard to drug options. This is valid independent of the clinical spectrum of the manifestations or even subtypes of the disease (46).

Exclusion criteria were as follows: pregnancy, breastfeeding, administration of biological or antibiotic therapy within 6 months prior to inclusion, nonauthorization for study participation, background of autoimmune diseases, malignancy, immunodeficiency, chronic pancreatitis, or chronic liver disease. Patients were classified as ReA (47) or uSpA (48) according to the ESSG classification criteria (45).

The sample size to estimate a 45% correlation was calculated, taking into account the standard deviation (SD) reported in the literature of the serum levels of SIgA in the healthy population (49–52), a confidence level (*Z*) of 95%, and an absolute precision (*d*) of 0.05. The population of patients classified as having uSpA and ReA, reported in the cohort of patients with SpA of the institution, were taken as a reference (53). The nonprobabilistic sampling of convenience was performed by nonrandomized selection of the available patients who attended the SpA clinic between November 2012 and May 2014. Of the 160 patients initially reported, 75 patients were excluded due to the use of biological therapy in the preceding 6 months, 24 were excluded due to the use of antibiotic therapy in the preceding 6 months, 10 were excluded due to the presence of neoplastic and/or autoimmune disease, and 5 were excluded for not signing the informed consent form. Control subjects were selected from the healthy residents of Bogotá, hospital visitors, and/or neighbors who voluntarily accompanied patients participating in the study. These controls could present unclassifiable osteomuscular symptoms as secondary to an autoimmune or autoinflammatory disease and were subject to the same exclusion criteria outlined above. Finally, 46 and 53 age-matched patients and controls, respectively, were included in the study.

Selected patients were questioned about demographic characteristics (age, sex, occupation, housing conditions, marital status, and education level); in addition, height and weight were measured. The clinical history was reviewed to find evidence of gastrointestinal symptoms (diarrhea, stools with mucous, hematochezia, number of stools per day, abdominal pain, and abdominal bloating), osteomuscular symptoms (lower back pain, arthritis, dactylitis, and enthesitis), history of uveitis, psoriasis, IBD, and HLA-B27 allele positivity. In addition, the period from the first symptom of the disease and the activity of the disease were calculated using the BASDAI, ASDAS-CRP, and ASDAS-ESR activity indices (49). At the same time, trained laboratory personnel withdrew and processed the serum samples for measuring SIgA, total IgA, specific anti-*C. trachomatis* and *Shigella* spp. IgA, ESR, and CRP from all the participants, with no knowledge of the results of the clinical evaluation.

### Variables of Analysis

#### ELISA Quantification of SIgA Antibodies in Serum

An in-house sandwich ELISA was developed as previously described (49, 50). A flexible, U-bottom ELISA plate was covered with an anti-secretor component (monoclonal anti-human SC, SIGMA, Cat No. I-6635), by placing 70 µl of the component in each well, at a dilution of 1/10,000 in phosphate-buffered saline (PBS) (54–56). As a negative control, wells covered in PBS incubated at 4°C overnight were used. After this time, the contents of the wells were eliminated, and plates were blocked with 150 µl per well of BLOTTO (nonfat dry milk with nonsterile PBS at a weight/volume concentration of 5%, plus TWEEN 20 at 0.1%) (54, 55); incubation was carried out for 50 min at 37°C.

Serial dilutions of serum samples from patients and healthy volunteers were carried out in 2.5% BLOTTO. To establish the standard calibration curve of the technique, purified SIgA derived from colostrum (purified SIgA from human colostrum AbD Serotec, Oxford, UK) was used. After incubation, the BLOTTO was discarded, and 70 µl of serum samples in 2.5% BLOTTO per well was dispensed, and incubated for 1 h and 45 min at 37°C. Three washes were then carried out with PBS-TWEEN 20 at 0.1%. Subsequently, 70 µl per well of anti-IgA biotin (KPL 16-10-01) at a 1/1,000 dilution in 2.5% BLOTTO was dispensed and incubated again for 50 min at 37°C. Three washes with PBS-TWEEN 20 at 0.1% were carried out and 70 µl per well of streptavidin peroxidase 1/1,000 was added (peroxidase-labeled streptavidin KPL 3000) and incubated with 50 min at 37°C. This was followed by three washes with 0.1% PBS-TWEEN 20. Finally, the reaction was revealed by the addition of 100 µl per well of TMB-H_2_O_2_ 1:1 (TMB Microwell Peroxidase Substrate System KPL 50-76-00), and the reaction was stopped with 17.5 µl per well of sulfuric acid 1 N when the sensitivity control reached a color intensity corresponding to an optical density of 0.1. Readings were performed with an ELISA-Thermo Lab System Multiskan EX reader at a wavelength of 450 nm.

#### Quantification of Total IgA in Serum with Kinetic Nephelometry

The total IgA levels were determined by kinetic nephelometry using the IMMAGE immunochemistry system from Beckman Coulter (Kit IMMAGE^®^ Immunochemistry Systems Chemistry Information Sheet IgA Ref. 446460) according to the manufacturer’s instructions. The biological reference interval for adults is 82–453 mg/dl.

#### ELISA for Determining the Final Titration of Specific IgA Antibodies for *Shigella* spp.

Flat-bottomed polystyrene plates (reference 120338, flat bottom polystyrene) were used, covered with sonicated bacteria (*Shigella flexneri* ATCC 12022) at a concentration of 20 µg/dl in 0.05 M sodium carbonate buffer (pH 9.6) and incubated overnight at 4°C. Plates were washed twice with PBS 1x-TWEEN 20 at 0.1% (PBST), and blocked with 200 µl of nonfat milk at 5% and PBS + Tween 20 at 0.1% (BLOTTO 5%), followed by incubation at 37°C for 50 min. Then, the contents of the plate were discarded and 100 µl per well of the serum samples was added, diluted at 1:100, 1:200, 1:400, and then incubated for 1 h and 45 min. As a positive control of the technique, purified total IgA (10 µg/ml of purified IgA from SIGMA) was used, while a sample of plasma derived from umbilical cord blood was used as a negative control.

Plates were then washed four times with PBST, and 100 µl of anti-IgA biotin (KPL 16-10-01) at a dilution of 1/1,000 in 2.5% BLOTTO was placed in each well and incubated for 50 min at 37°C. Plates were then washed four more times with PBST, and 100 µl of streptavidin peroxidase (peroxidase-labeled streptavidin KPL 143000) at a dilution of 1/1,000 in 2.5% BLOTTO was placed in each well and incubated for 45 min at 37°C. This was followed by four washes with PBST. Finally, the reaction was revealed by using 100 µl of TMB-H2O2 1:1 (TMB Microwell Peroxidase Substrate System KPL 50-76-00) per well. The reaction was stopped with 50 µl per well of 2.5 N sulfuric acid when the sensitivity control of the IgA curve reached a color intensity corresponding to an optic density of 0.1. Readings were performed with the ELISA Lector de MicroELISAS Tecan Infine F200/M200 reader, at a wavelength of 450 nm.

#### ELISA for Evaluating Serum Levels of IgA Antibodies Specific to *C. trachomatis*

Hemolyzed and lipemic samples [DIA.PRO Diagnostic Bioprobes Srl-Ref CTA.CE, San Giovanni (Milan), Italy] were rejected. Samples were centrifuged at 2,000 rpm for 20 min and stored at −20°C until required for processing.

Microplates were covered in specific polypeptide antigens derived from the external membrane of *C. trachomatis*. The solid phase was treated with the sample diluted at 1:101, with the exception of controls and anti-IgA antibodies against *C. trachomatis* marked with horseradish peroxidase (HRP). Microplates were then washed. A volume of 100 µl of sulfuric acid was then added to each well. Finally, the color intensity of the solution in each well was measured at a wavelength of 450 nm for reading in the filter and at 620–630 nm for equipment calibration. A positive result was considered under 1.0.

#### Determination of the HLA-B27 Allele Flow Cytometry

The qualitative determination of HLA-B27 in the total blood of the patients was performed by flow cytometry using BD FACSCanto™ (BD^TM^ HLA-B27 KIT Ref. 340183 Becton Dickinson, San José, CA) according to the manufacturer’s instructions, together with analysis using FCAP array software, Windows version.

#### Determination of High-Sensitivity CRP

We used the chemiluminescence (Immulite 1000, Siemens^®^) REF LKCRP1 kit, according to the manufacturer’s instructions; reference values were 0–3 mg/dl.

#### Determination of ESR

This was performed with an automated method of quantitative capillary photometry in millimeter per hour according to the manufacturer’s instructions.

### Statistical Analysis

For continuous variables, central tendency and variability were calculated. For the categorical variables, proportions were calculated. A correlation analysis was carried out using the Pearson correlation coefficient; comparisons for nonrelated variables were performed using the Student’s *t*-test, and inter-groups comparisons were made by Kruskal–Wallis with *U* Mann–Whitney test correction. A confirmatory multivariate evaluation by principal components analysis (PCA) was made. Categorical variables were analyzed using the Chi-squared test or Fisher’s exact test, as appropriate. Linear regression models were used to evaluate the association between disease activity and the serum levels of SIgA. Statistical significance was defined as a *p*-value <0.05. All analysis were made using Stata version 11.2^®^ for Windows, R V3.3.21.

## Results

### Sociodemographic and Clinical Characteristics

This study included 46 patients with ReA and uSpA: 78.2% were male, with a mean age of 34.8 (SD, 12.3) years and body mass index (BMI) >25 was 30.4%. Of the 53 healthy controls, 41% were male, with a mean age of 32 (SD, 11.4) years and BMI >25 was 30.1%.

Of the SpA patients, 69% reported the presence of some symptoms of gastrointestinal origin. The most frequent gastrointestinal complaints were abdominal bloating, abdominal pain, and more than two stools per day. All patients reported at least one osteomuscular complaint, with the most frequent being enthesitis, inflammatory lower back pain, and arthritis. Fatigue was reported in 62% of patients, and 44% of patients reported a previous infection, 6% of the patients smoked, 4.3% had a family history of SpA, and 47.8% were HLA-B27-positive. At the time of enrollment, 47% of the patients had 12 months or less of disease duration, and in most of the patients, the status of the disease was reported with the disease activity measurements (BASDAI ≥ 4: 63.6%; ASDAS-PCR ≥ 2.1: 68.1%; ASDAS-VSG ≥ 2.1: 82.1%) (Table 1).

**Table 1:** **Clinical variables of patients**.

Variables	Patients (*n* = 46)	
	*F*	%
Any gastrointestinal symptoms	29	69.00
Diarrhea	9	21.40
>2 depositions day	17	41.40
Hematochezia	6	14.20
Mucus in the stool	5	11.90
Abdominal pain	18	42.80
Abdominal distention	19	45.20
Any musculoskeletal symptoms	46	100.00
Inflammatory back pain	29	63.00
Arthritis	27	58.60
Enthesitis	32	69.50
Dactylitis	9	19.50
**Other symptoms**		
Weight loss >5%/last month	12	28.50
Fatigue	26	61.90
**Extra-articular symptoms**		
Uveitis	7	15.20
Psoriasis	0	0.00
Inflammatory bowel disease (IBD)	0	0.00
**Symptoms time (months)**		
Mean ± SD	33.8 ± 41.6	
Median (min–max)	18.5 (1–192)	
≤12 months	22	47.80
>12 months	24	52.10
HLA-B27^+^	22	47.80
HLA-B27^−^	24	52.20
**Background**		
Family history	2	4.30
Previous infection (G/I G/U)	20	43.40
Smoking	6	13.00
BASDAI, mean ± SD	5.3 ± 2.6	
Inactive disease (<4)	16	36.40
Active disease (≥4)	28	63.60
ASDAS-CRP, mean ± SD	2.7 ± 1.2	
Inactive disease	5	11.30
Moderate disease activity	9	20.40
High disease activity	20	45.40
Very high disease activity	10	22.70
ASDAS-ESR, mean ± SD	3.1 ± 1.1	
Inactive disease	0	0.00
Moderate disease activity	8	17.70
High disease activity	21	46.60
Very high disease activity	16	35.50

*ASDAS, Ankylosing Spondylitis Disease Activity Score; BASDAI, Bath Ankylosing Spondylitis Disease Activity Index; CRP, C-reactive protein; ESR, erythrocyte sedimentation rate; *F* (%), frequency and percentage*.

### Levels of SIgA, Total IgA, and Specific IgA (*Shigella* spp., *C. trachomatis*) in Serum

In SpA patients, the mean concentration of serum SIgA was 19.85 ± 9.97 µg/ml, whereas in healthy controls, the SIgA serum level was 10.82 ± 6.5 µg/ml (*p* < 0.001). The statistical significance of difference was maintained independently of the presence of the HLA-B27 allele and the length of symptoms of the patients (Table 2).

**Table 2:** **Comparison of serum concentrations of SIgA, total IgA, IgA anti-*Chlamydia trachomatis* antibodies, and IgA anti-*Shigella* spp. antibodies between patients and controls**.

Variable	Controls (*n* = 53)	Patients				
		Total, *n* = 46 (*p* value)	HLA-B27^+^, *n* = 22 (*p* value)	HLA-B27^−^, *n* = 24 (*p* value)	>12 m, *n* = 24 (*p* value)	≤12 m, n = 22 (*p* value)
**SIgA (g/ml)**						
Mean ± SD	10.8 ± 6.5	19.8 ± 9.9 (<0.001)^a^^,^^†^	19.5 ± 11.5 (0.0001)^a^^,^^†^	20.1 ± 8.6 (<0.001)^a^^,^^†^	22.9 ± 10.7 (0.001)^a^^,^^†^	16.7 ± 8.2 (0.0015)^a^^,^^†^
**Total IgA (mg/dl)**						
Mean ± SD	284 ± 107	275 ± 123 (0.72)^a^	299 ± 140 (0.62)^a^	255 ± 106 (0.27)^a^	292.7 ± 132 (0.76)^a^	257.9 ± 115 (0.349)^a^
**IgA anti-***C. trachomatis*** (ratio)**						
Median [min–max]	0.44 [0.15–1.75]	0.55 [0.12–13.03] (0.06)^b^	0.46 [0.12–13.03]	0.59 [0.29–10.4]	0.67 [0.11–3.57]	0.45 [0.17–13.0]
**IgA anti-***Shigella*** spp. (titers)**						
Median [min–max]	1/800 [1/200–1/6,400]	1/800 [1/200–1/6,400] (0.12)^b^	1/400 [1/200–1/6,400]	1/800 [1/200–1/3,200]	800 [1/200–1/6,400]	800 [1/200–1/6,400]

*^a^Student’s t-test*.

*^b^Mann–Whitney test*.

*^†^Statistical significance, p < 0.05*.

*HLA-B27^+^, HLA-B27 allele positive; HLA-B27^−^, HLA-B27 allele negative; SIgA, secretory immunoglobulin A; >12 m, more than 12 months from diagnosis; <12 m, less than 12 months from diagnosis*.

No differences were found in the mean concentration of serum SIgA in relation to the status of the HLA-B27 allele (*p* = 0.83) in SpA patients. However, there were statistically significant differences in relation to the length of symptoms (*p* = 0.036).

There was an association between the presence of abdominal bloating and serum levels of SIgA below 15.79 µg/ml, *r* = 0.69 (*p* = 0.049). This association was maintained in patients with an evolution time of ≤12 months (*p* = 0.002). No association was observed in the levels of SIgA with respect to osteomuscular symptoms, fatigue, or medical history (data not shown).

The mean concentration of total IgA in patients was 275 ± 123 mg/dl, and in healthy controls, it was 284.33 ± 107 mg/dl. This difference was not statistically significant, and the difference was not affected by the HLA-B27 status or the disease duration (Table 2).

In the SpA patients, there were no statistically significant differences in the average concentration of total IgA in relation to the status of the HLA-B27 allele (*p* = 0.23). The same was observed in relation to the length of symptoms (*p* = 0.35).

A total of 11 SpA patients had positive anti-*C. trachomatis* IgA antibodies (26.1%) and 6 had titers of anti-*Shigella* spp. IgA greater than 1/1,600 (13.3%); there were no statistically significant differences between the median serum level of anti-*C. trachomatis* IgA (0.55 vs. 0.44; *p* = 0.06) and the titers of anti*-Shigella* spp. IgA (1/800 vs. 1/800, *p* = 0.12) between patients and controls (Table 2).

Among patients, with relation to the status of the HLA-B27 allele and the length of symptoms of the disease, there were no statistically significant differences in the median serum levels of anti-*C. trachomatis* IgA (HLA-B27^+^ vs. HLA-B27^−^, *p* = 0.20) (≤12 vs. >12 months, *p* = 0.37) or the titers of anti-*Shigella* spp. IgA (HLA-B27^+^ vs. HLA-B27^−^, *p* = 0.24) (≤12 vs. >12 months, *p* = 0.98).

No association was found between the serum levels of the different molecular forms of IgA by chi square test. There was also no correlation between the different molecular forms of IgA and serum inflammatory markers. The exception was with the ESR and anti-*C. trachomatis* IgA (*r* = 0.32, *p* = 0.04), which presented a low, statistically significant, positive correlation.

No association was found between the composite activity indices and the levels of total IgA, anti-*C. trachomatis* IgA, and the titers of anti-*Shigella* spp. IgA.

**Figure 1:**
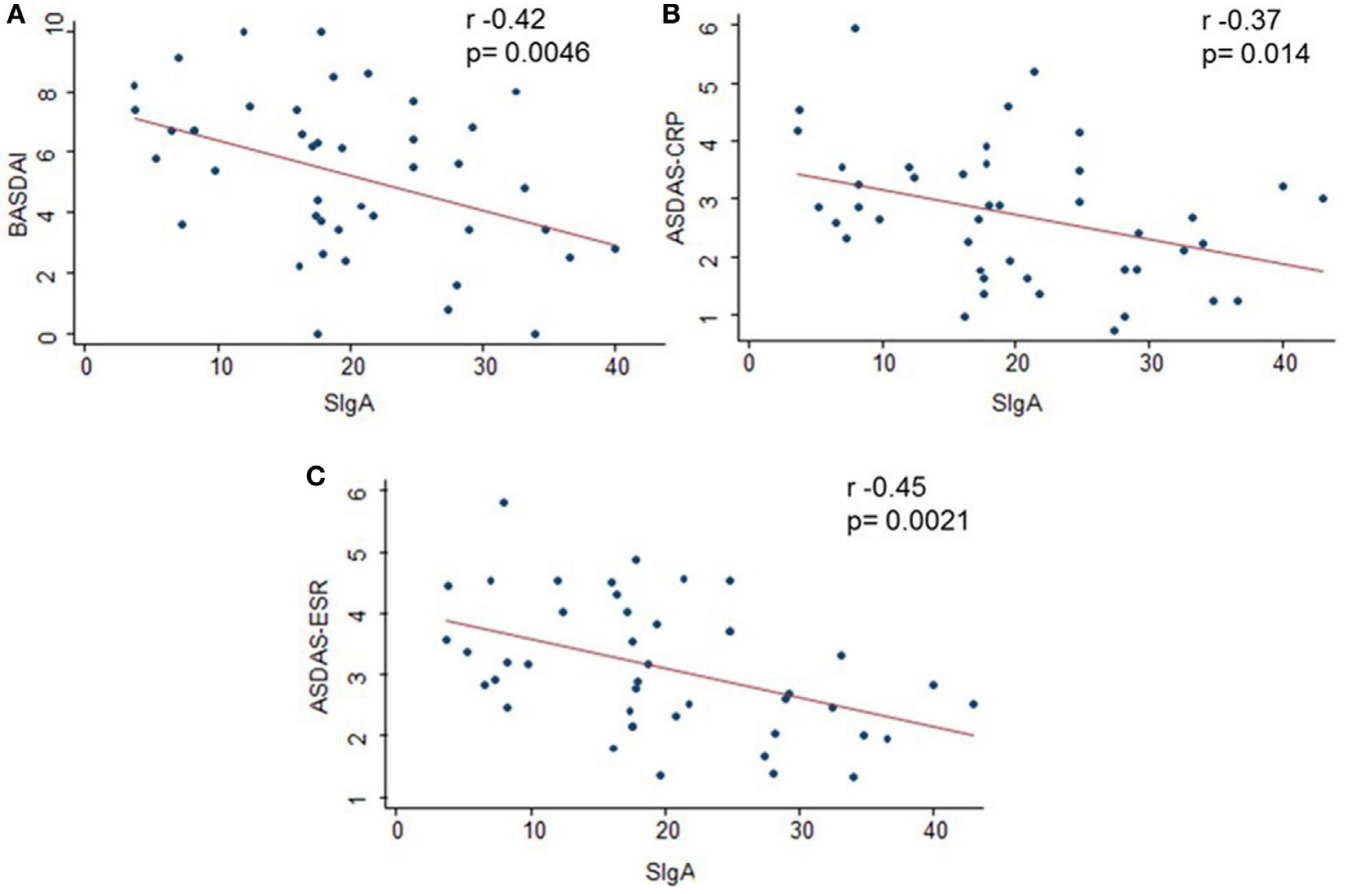
**Correlation between serum secretory immunoglobulin A (SIgA) and clinical index**. A moderate negative correlation was found between SIgA and the disease activity measurements. Correlations were evaluated by Pearson tests. A *p* value of <0.05 was considered statistically significant. **(A)** Negative correlation between Bath Ankylosing Spondylitis Disease Activity Index and SIgA (*p* = 0.0046; *r* = −0.42). **(B)** Negative correlation between Ankylosing Spondylitis Disease Activity Score (ASDAS)-C-reactive protein (CRP) and SIgA (*p* = 0.014; *r* = −0.37). **(C)** Negative correlation between ASDAS-erythrocyte sedimentation rate (ESR) and SIgA (*p* = 0.0021; *r* = −0.45). *x*-axis: SIgA in serum (micrograms per milliliter).

The disease activity measurements had a moderate negative correlation with serum levels of SIgA: BASDAI and SIgA (*r* = −0.42, *p* = 0.0046), ASDAS-CRP and SIgA (*r* = −0.37, *p* = 0.014), ASDAS-ESR and SIgA (*r* = −0.45, *p* = 0.0021) (Figure 1); these negative correlations were stronger in the subgroup of patients who were HLA-B27-positive: BASDAI and SIgA (*r* = −0.70, *p* = 0.0009), ASDAS-CRP and SIgA (*r* = −0.58, *p* = 0.0093), ASDAS-ESR and SIgA (*r* = −0.57, *p* = 0.0083), and also when analyzing patients with length of symptoms greater than 12 months: BASDAI and SIgA (*r* = −0.53, *p* = 0.01), ASDAS-ESR and SIgA (*r* = −0.55, *p* = 0.006) (Table 3).

**Table 3:** **Association between disease activity measurements and serum levels of SIgA, total IgA, IgA anti-*Chlamydia trachomati*s antibodies, and IgA anti-*Shigella* spp. antibodies**.

	BASDAI *r* (*p* value)	ASDAS-CRP *r* (*p* value)	ASDAS-ESR *r* (*p* value)	SIgA *r* (*p* value)	IgA total *r* (*p* value)	IgA anti-*C. trachomatis *r* (*p* value)*	IgA anti-*Shigella* spp. *r* (*p* value)
BASDAI	1						
ASDAS-CRP	0.69 (<0.001)^†^	1					
ASDAS-ESR	0.80 (<0.001)^†^	0.84 (<0.001)^†^	1				
SIgA	−0.42 (0.0046)^†^	−0.37 (0.014)^†^	−0.45 (0.0021)^†^	1			
IgA total	−0.19 (0.21)	−0.1 (0.95)	−0.15 (0.32)	0.18 (0.074)	1		
IgA anti-*C. trachomatis*	0.13 (0.4)	0.13 (0.42)	0.27 (0.08)	0.16 (0.13)	0.5 (0.63)	1	
IgA anti-*Shigella* spp.	0.15 (0.33)	0.14 (0.35)	0.12 (0.44)	−0.17 (0.09)	−0.13 (0.22)	0.4 (0.69)	1

*^†^Statistical significance, p < 0.05*.

*Pearson correlation coefficient, r (p value)*.

*ASDAS, Ankylosing Spondylitis Disease Activity Score; BASDAI, Bath Ankylosing Spondylitis Disease Activity Index; CRP, C-reactive protein; ESR, erythrocyte sedimentation rate; SIgA, secretory immunoglobulin A*.

**Figure 2:**
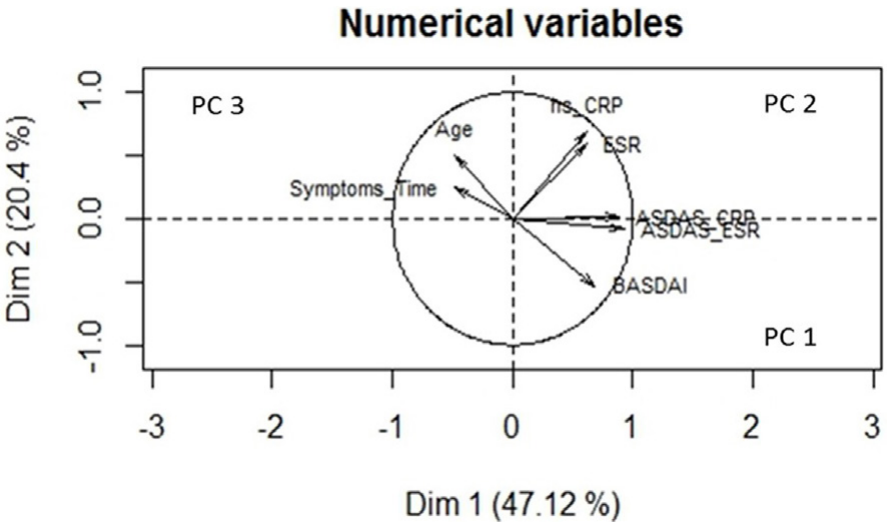
**Variables vector map—principal component analysis (PCA)**. This geometric representation of the variables is presented by arrows of a length equal to the standard deviation of the variable. The angle between each pair of arrows represents the correlation between these two variables. Small angles mean a high positive correlation, angles close to 90° no correlation, and angles close to 180° a high negative correlation with opposite directions in the plane. The PCA graph displays the graphical representation of the analyzed components matrix. From the representation, it is extracted that the explanation of the underlying factors that could somehow influence the secretory immunoglobulin A (SIgA) levels together: the first component (PC1) is a factor that represents variables associated with the evaluation of the clinical activity of the disease: Ankylosing Spondylitis Disease Activity Score (ASDAS)-C-reactive protein (CRP), ASDAS-erythrocyte sedimentation rate (ESR), and Bath Ankylosing Spondylitis Disease Activity Index (BASDAI). The second component (PC2) is a factor which group serological variables that are considered soluble markers of activity of the disease and the third component (PC3) aggregates two independent variables as they are age and symptoms that could modify the levels of SIgA. Dim, dimensions.

To confirm the latest results with a method more accurate, PCA was performed, it is a technique used to emphasize variation and bring out strong patterns in a dataset. It is often used to make data easy to explore and visualize. The PCA method achieve the analogical presentation of the information using geometric principles that studies, among others, the relationships between the variables using canonical Euclidean distance (56). The results showed three principal factors that cover a contribution of 82.1% to explain the SIgA variability. The first component (PC1) was constituted by ASDAS-CRP, ASDAS-ESR, and BASDAI variables, which provide the 47.1% refer as clinical activity measures. The PC2 did include the serological activity markers CRP and ESR with a weight of 20.4%, and the last component PC3 was compounded by age and symptoms time which contribute the 14.6% (Figure 2).

We performed a simple linear regression model, looking for any correlation between disease activity measurements and serum levels of SIgA. The correlation was statistically significant and was stronger for BASDAI and SIgA (−0.12, *p* = 0.005), compared to ASDAS-CRP and SIgA (−0.04, *p* = 0.014) or ASDAS-ESR and SIgA (−0.048, *p* = 0.002). We found that for every 1 µg/ml increase of SIgA, the BASDAI disease activity measurements decreased by about 0.12 units, the ASDAS-CRP decreased by about 0.4 units, and the ASDAS-ESR decreased by about 0.048 units (see Eqs 1–3).

(1) BASDAI=7.56−0.12×SIgA(μg/ml)

(2) ASDAS−PCR=3.6−0.04×SIgA(μg/ml)

(3) ASDAS−VSG=4.05−0.048×SIgA(μg/ml)

When the linear regression model was stratified, an increase in the regression coefficient in the three correlations among the HLA-B27-positive patients was observed. These findings were consistent in patients with length of symptoms more than 12 months and remained statistically significant (Table 4).

**Table 4:** **Simple and stratified linear regression models to show the correlation of disease activity measurements and SIgA levels**.

Models	Crude association (*p* value)	Stratified association (*p* value)			
		HLA-B27^+^	HLA-B27^−^	Symptoms time: ≤12 months	Symptoms time: >12 months
BASDAI vs. SIgA	−0.12 (0.005)^†^	−0.14 (0.004)^†^	−0.07 (0.34)	−0.07 (0.346)	−0.12 (0.01)^†^
ASDAS-CRP vs. SIgA	−0.04 (0.014)^†^	−0.055 (0.02)^†^	−0.02 (0.36)	−0.04 (0.23)	−0.03 (0.13)
ASDAS-ESR vs. SIgA	−0.048 (0.002)^†^	−0.053 (0.013)^†^	−0.04 (0.099)	−0.03 (0.33)	−0.04 (0.006)^†^

*^†^Statistical significance, p < 0.05*.

*ASDAS, Ankylosing Spondylitis Disease Activity Score; BASDAI, Bath Ankylosing Spondylitis Disease Activity Index; CRP, C-reactive protein; ESR, erythrocyte sedimentation rate; SIgA, secretory immunoglobulin A*.

## Discussion

The main objective of this study was to explore whether there is a correlation among the serum levels of SIgA, total IgA, IgA anti-*C. trachomatis*, and IgA anti-*Shigella* spp. with the disease activity measures and acute phase reactants in patients with SpA. We found that higher serum levels of secretory IgA were correlated with low disease activity parameters in patients with SpA.

Circulating SIgA has been detected in the serum of healthy individuals regardless of the sex or age group (48, 49); however, the mechanism by which SIgA, produced at mucosal surfaces, is transported to the circulation remains to be determined (57). Recently, it was proposed that Dectin-1 is essential for the retrotranscytosis of glycosylated SIgA-antigen complexes by intestinal M cells (58) and the CD71 receptor (59). The low serum concentration of SIgA was probably due to the subepithelial dome in the lamina propia after retrotranscytosis; however, the mechanism by which SIgA is transported to the circulation and the physiological function of SIgA in the systemic compartment are unknown (60). To date, the mechanisms of aberrant transcytosis at the wrong side of the epithelium are not known.

Many studies have suggested that mucous membranes and their immune system play a physiopathological role in the development and maintenance of many autoinflammatory diseases including SpA (61). The mean serum levels of SIgA that we found in the control population are similar to data reported in the literature by Delacroix and Vaerman (51) and Kvale and Brandtzaeg (52). In agreement with previous studies (17, 41), our data reveal a higher mean concentration of SIgA in patients with ReA and uSpA, independently of the status of the HLA-B27 allele and the length of disease duration. In the study by Granfors (18), there was a statistically significant difference in the serum levels of SIgA, and specifically anti-*Y. enterocolitica* SIgA levels in patients who developed arthritis after a confirmed infection. Similarly, Wendling et al. (42) reported higher serum levels of SIgA and free SC in patients with AS. These similar findings in different study populations suggest a higher production of Ig, characteristic of the mucosal immune system, and imply that high serum levels of SIgA represent the humoral compensatory mechanism provided by the mucosal immune system when faced with a greater exposure to microbial products of the intestinal lumen (42, 62).

Likewise, the SIgA has been implicated in the pathophysiology of certain diseases, for example, Rotavirus (RV)-specific SIg has been previously detected in the serum of naturally RV-infected children, and was shown to reflect the intestinal Ig immune response (55). Elevated levels of SIgA have also been documented in patients with HIV infection (63) and alcoholic liver disease (64). In patients with IgA nephropathy, the level of serum SIgA has been shown to correlate with the severity, suggesting a role for SIgA in disease pathogenesis (65). The reports, in this regard, on IBD are scarce. In 1987, a study in serum SIgA measurement was carried out without significant differences being obtained against controls and without any association with the severity and activity of pathologies (66). Some studies have been carried out to determine if there are any alterations in the production of SIgA in individuals with IBD, wherein a decrease in IgA-producing plasma cells has been in patients with Crohn’s disease (67). Otherwise, in the mucosa of patients with ulcerative colitis (UC) without increase in SIgA levels, suggesting a defect in the transcytosis process of IgA (68). The SC can also be seen as an essential constituent of SIgA, and the aforementioned clinical associations have been found with SIgA and not with free SC (63–65).

Levels of anti-*C. trachomatis* IgA were analyzed, in both patients and controls. In SpA patients with anti-*C. trachomatis* IgA antibodies, all had detectable serum levels of SIgA, and 20% had IgA levels greater than the reference value. In contrast, in the control group with anti-*C. trachomatis* IgA antibodies, none had elevated SIgA or total IgA serum levels. The data from these populations reflect a pattern of differential humoral immune response after exposure to *C. trachomatis* antigens. Bas et al., in 2001 (69), reported that in patients with acute urogenital infection from response due to a lower number of antigenic targets for IgA antibodies. We are unaware of any studies that explore the production of SIgA and total IgA in the serum of patients infected by C. *trachomatis* and its relationship with the development of ReA. To address this question, a study with a larger population is required.

No association was found between serum levels of SIgA, total IgA, and specific IgA: anti-*C. trachomatis* IgA or anti*-Shigella* spp. IgA. This suggests that the SIgA and total IgA produced by the humoral effector of mucosal and systemic immunity, respectively, can reflect the independent behavior of these two systems. This lack of association had been previously described by Thompson and Asquith in 1970 after not finding any correlation between the serum levels of SIgA and total IgA, given their synthesis source (50); likewise, Wendling et al. (42) did not find a correlation between SIgA and IgA in patients with AS; and Granfors (18) did not report a correlation between the two immunoglobulins in patients with ReA.

In our study, the PCA multivariate analysis allowed to establish the relationship between the serum inflammatory markers (high sensitivity CRP and the ESR) and the serum levels of SIgA was observed in uSpA and ReA. In 1996, Wendling et al. (42) reported similar results between SC and CRP levels in patients with AS.

Evaluating the association among the clinical manifestations, background, and the categorized levels of SIgA, an association between lower serum concentration of SIgA and the presence of abdominal bloating (as a gastrointestinal complaint) was observed. This could suggest the importance of the protective effect of this type of Ig in the mucosal immune system (31).

Finally, we found a moderate correlation between the clinical disease activity indices (BASDAI, ASDAS-CRP, ASDAS-ESR) and the serum concentration of SIgA. These negative correlations were stronger in the HLA-B27-positive subgroup and in patients with length of symptoms greater than 12 months.

The strength of the association between the composite activity indices and the serum concentrations of SIgA was confirmed with linear regression models and PCA. In addition, the greatest strength of this association was in the subgroup of HLA-B27-positive patients. The data presented above demonstrate that the increase in the serum concentration of SIgA translates into a better clinical control of the disease, supporting our hypothesis that the serum concentration of SIgA could modulate the clinical status of patients with ReA and uSpA.

This study has limitations. The methodology design of this cross-sectional study does not allow us to clearly establish a time relationship between the serum concentration of SIgA and low disease activity in SpA. Furthermore, there is a need for follow-up and monitoring these patients to investigate the dynamics of changes in humoral response. Additionally, this follow-up may provide us evidence to link whether persistent high level of total IgA may help to modulate the progression of the disease toward a structural sacroiliac involvement or developing extensive axial inflammation as suggested by Franssen and collaborators (38).

The relatively low prevalence and incidence of SpA in the general population, as well as its chronicity and insidious onset, caused us to carry out a nonprobabilistic sampling, due to convenience, which is one of the main limitations of this study. This potential Berkson’s bias would facilitate the participation in the study of patients who had previously accessed specialized medical consultations more frequently and, therefore, probably included patients with the highest disease activity. The sample size, despite being small, was large enough to establish a relationship between the variables of the study. In terms of the measurement bias, it is necessary to stress the importance of the standardization of tests for measuring the serum levels of the different types, subtypes, and forms of serum immunoglobulins. Finally, there is a potential memory bias from asking the patients about clinical variables; however, this was minimized by comparing the collected information with clinical medical histories.

In conclusion, this study suggests that higher SIgA levels are correlated with low disease activity parameters in patients with ReA and uSpA and based on the status of the HLA-B27 allele. These findings may reflect a possible immunomodulatory effect of this potential biomarker with implications in clinical practice.

## Ethics Statement

The study complied with the ethical principles for medical research on human beings established in the Helsinki Declaration of the World Medical Association (WMA) and in compliance with the scientific, technical, and administrative regulations for health research established in resolution No. 8430 of 1993 enacted by the Ministry of Health of Colombia. In addition, the study was approved by the Ethics and Research Committee of the Hospital Militar (No. C-2012-078), and all patients sign the informed consent.

## Author Contributions

FS-C and CR-S contributed to the conception or design of the work; the acquisition, analysis, and interpretation of data for the work; drafting the work and revising it critically for important intellectual content; and final approval of the version to be published. JB-G, WB-M, IA, DH, DC, RV-O, and LC-M contributed to the acquisition of data for the work, revising the manuscript, and final approval of the version to be published.

## Conflict of Interest Statement

The authors declare that the research was conducted in the absence of any commercial or financial relationships that could be construed as a potential conflict of interest.
